# Mutant *Enpp1^asj^* mice as a model for generalized arterial calcification of infancy

**DOI:** 10.1242/dmm.012765

**Published:** 2013-06-20

**Authors:** Qiaoli Li, Haitao Guo, David W. Chou, Annerose Berndt, John P. Sundberg, Jouni Uitto

**Affiliations:** 1 Department of Dermatology and Cutaneous Biology, Jefferson Medical College, Thomas Jefferson University, Philadelphia, PA 19107, USA; 2 Division of Pulmonary, Allergy and Critical Care Medicine, Department of Medicine, University of Pittsburgh, PA 15213, USA; 3 The Jackson Laboratory, Bar Harbor, ME 04609, USA; 4 Department of Biochemistry and Molecular Biology, Jefferson Medical College, Thomas Jefferson University, Philadelphia, PA 19107, USA; 5 Jefferson Institute of Molecular Medicine, Thomas Jefferson University, Philadelphia, PA 19107, USA

## Abstract

Generalized arterial calcification of infancy (GACI), an autosomal recessive disorder, is characterized by early mineralization of blood vessels, often diagnosed by prenatal ultrasound and usually resulting in demise during the first year of life. It is caused in most cases by mutations in the *ENPP1* gene, encoding an enzyme that hydrolyzes ATP to AMP and inorganic pyrophosphate, the latter being a powerful anti-mineralization factor. Recently, a novel mouse phenotype was recognized as a result of ENU mutagenesis – those mice developed stiffening of the joints, hence the mutant mouse was named ‘ages with stiffened joints’ (*asj*). These mice harbor a missense mutation, p.V246D, in the *Enpp1* gene. Here we demonstrate that the mutant ENPP1 protein is largely absent in the liver of *asj* mice, and the lack of enzymatic activity results in reduced inorganic pyrophosphate (PP_i_) levels in the plasma, accompanied by extensive mineralization of a number of tissues, including arterial blood vessels. The progress of mineralization is highly dependent on the mineral composition of the diet, with significant shortening of the lifespan on a diet enriched in phosphorus and low in magnesium. These results suggest that the *asj* mouse can serve as an animal model for GACI.

## INTRODUCTION

Several mendelian genetic disorders have recently been shown to result in vascular mineralization, with profound phenotypic manifestations (Li and Uitto, 2013; Nitschke and Rutsch, 2012b). The prototype of such conditions is generalized arterial calcification of infancy (GACI), an autosomal recessive disorder characterized by early mineralization of blood vessels, often diagnosed prenatally through ultrasound (Rutsch et al., 2011). The newborns manifest with severe hypertension, cardiomyopathy and heart failure, resulting in demise of the affected individuals in most cases during the first year of life. GACI is caused by loss-of-function mutations in the *ENPP1* gene, which codes for ectonucleotide pyrophosphatase/phosphodiesterase 1 (ENPP1), an enzyme that hydrolyzes ATP to AMP and inorganic pyrophosphate (PP_i_) (Ruf et al., 2005). Because PP_i_ is a powerful local inhibitor of ectopic mineralization, in the absence of the ENPP1 activity, progressive vascular mineralization ensues.

A number of mouse models have been identified to recapitulate the features of human diseases with vascular mineralization (Li and Uitto, 2013; Mackenzie et al., 2012). A mutant mouse with a missense mutation (p.V246D) in the *Enpp1* gene was recently identified by the neuromutagenesis program at The Jackson Laboratory as a result of ENU treatment (http://mousemutant.jax.org/articles/mmrmutantasj.html). These mice demonstrated stiff posture, abnormalities in the front legs and stiffening of the joints. The standard pathological screen performed at 7 months of age revealed very stiff and unbendable joints with severe osteoarthritis; hence, this mutation was named ‘ages with stiffened joints’ (*asj*). An interesting histopathological observation in these mice was mineralization of the dermal sheath of vibrissae, an observation that we had previously made in *Abcc6^tm1Jfk^* knockout mice, a model for pseudoxanthoma elasticum (PXE), which develop late-onset mineralization of the dermis, arterial blood vessels and Bruch’s membrane in the eye (Klement et al., 2005). Considering the apparent overlap of aberrant mineralization between GACI and PXE, we have now carefully characterized the *Enpp1^asj^* mouse as a potential model for GACI.

## RESULTS

### Phenotypic manifestations of *Enpp1^asj^* mice

**Fig. 1.**
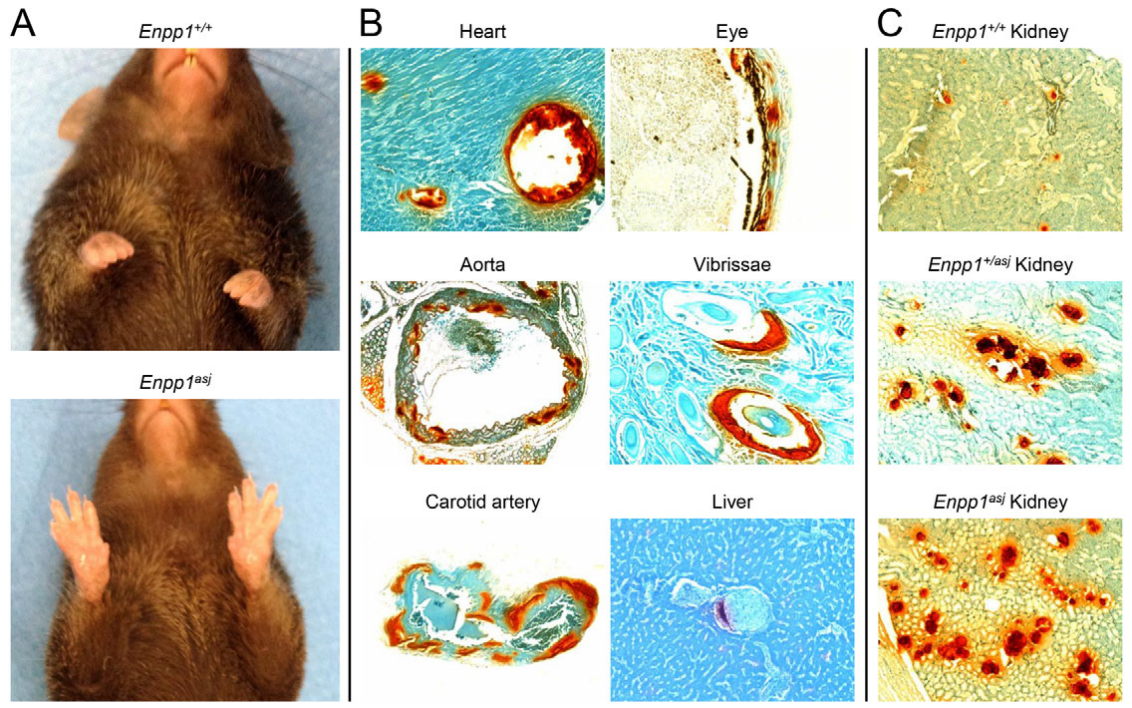
**Phenotypic presentation and aberrant mineralization in *asj* mice at 12 weeks of age.** (A) The *Enpp1^asj^* mice develop progressive stiffening of the joints leading to contractures as shown on the front paws (lower panel) in comparison with a corresponding wild-type mouse (upper panel). (B) Histopathology reveals extensive mineralization in the heart, aorta, carotid artery, retina of the eye and dermal sheath of vibrissae, but not in the liver parenchyma of *asj* mice. (C) Extensive aberrant mineralization in the kidneys of heterozygous (middle panel) and homozygous (lower panel) *asj* mice. Focal areas of mineralization are also noted in the kidneys of wild-type mice (upper panel). Alizarin red stain; original magnifications: heart, carotid artery, eye, vibrissae, liver, 150×; aorta, kidney, 100×.

The *Enpp1^asj^* mice were obtained from The Jackson Laboratory, and by ∼2 months of age they were noted to have stiffening of the joints, particularly the forepaws, which resulted in a slow, hobbling gait that worsened as they aged (Fig. 1A). This process was clearly accelerated when the mice were placed on an ‘acceleration diet’, rich in phosphorus and low in magnesium (Jiang and Uitto, 2012).

**Fig. 2.**
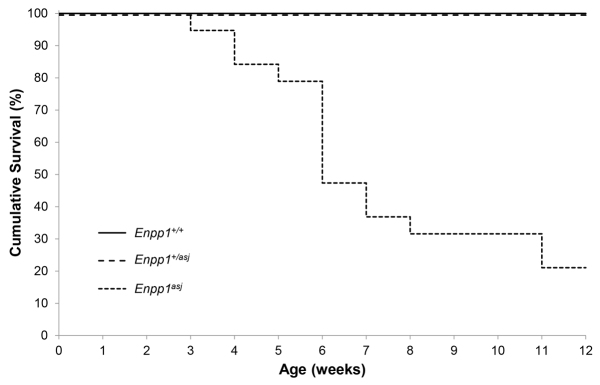
**Kaplan-Meier survival curves of *asj* mice on the acceleration diet.** Note that >50% of *asj* mice died spontaneously prior to age 6 weeks, whereas the heterozygous mice (*Enpp1^+/asj^*) had a survival similar to wild-type controls.

In spite of the limited locomotion, the *asj* mice that were kept on a normal laboratory diet had a normal lifespan. However, if the mothers were placed on the acceleration diet during pregnancy and the pups were placed on the same diet at weaning at 4 weeks of age, the lifespan of the mice was drastically reduced (Fig. 2). Specifically, more than 50% of the *asj* mice died spontaneously before the age of 6 weeks, and the average age of demise was 6.4±0.6 weeks (mean ± s.e.m.; *n*=15). There was no difference in the age of death between males and females (*P*=0.47; Student’s *t*-test). Only 4 out of 28 *asj* mice survived to 12 weeks of age; these mice were then sacrificed for analysis.

We also looked for evidence of embryonic lethality in the *asj* mice by genotyping a total of 136 newborn pups representing 35 litters from heterozygous mating pairs. The distribution between wild-type, heterozygous and homozygous *asj* mice was 37:71:28. This distribution did not differ from the expected mendelian distribution of 34:68:34 (χ^2^=1.456; *P*=0.483). Thus, the *asj* mutant mice have a significantly shortened lifespan when placed on a special diet, but there is no evidence of embryonic lethality.

**TRANSLATIONAL IMPACT**

**Clinical issue**

A number of heritable disorders manifest with aberrant mineralization of the skin and vascular connective tissues. These conditions are characterized by a broad spectrum of phenotypic variability and, in some cases, considerable morbidity and mortality. One such condition is generalized arterial calcification of infancy (GACI), which is diagnosed on the basis of prenatal or perinatal calcification of arterial blood vessels. Affected children usually die within the first year of life. Most individuals with GACI harbor mutations in the *ENPP1* gene, which encodes the enzyme ectonucleotide pyrophosphatase/phosphodiesterase 1 that hydrolyzes ATP to AMP and inorganic pyrophosphate (PP_i_). PP_i_ is a powerful local inhibitor of ectopic mineralization; thus, in the absence of ENPP1 activity, progressive vascular mineralization ensues. New animal models of GACI are needed to investigate the mechanisms involved in vascular mineralization and its associated disorders.

**Results**

A novel mouse model for GACI was recently identified as a result of ENU mutagenesis. A characteristic feature of these mice is stiffening of the joints that worsened with age; hence, the mouse phenotype was designated as ‘ages with stiffened joints’, *asj*. These mice develop progressive mineralization of the skin, aorta, coronary arteries, and arterial blood vessels in a number of tissues. In the present study, the *asj* mice were characterized further. The development of the mineralization phenotype could be noted soon after birth when the mice were placed on an ‘acceleration diet’, rich in phosphorus and low in magnesium. The underlying molecular defect was demonstrated to be a homozygous T-to-A transversion mutation in position 771 of the *Enpp1* gene resulting in homozygous p.V246D substitution. The mutant allele resulted in normal levels of the corresponding mRNA transcript, but the level of ENPP1 protein was below the detection limit by western analysis, and the ENPP1 enzymatic activity was reduced to <20% of the corresponding wild-type mouse. As a result of reduced ENPP1 enzymatic activity, the PP_i_:P_i_ ratio was markedly reduced. The heterozygous *Enpp1^+/asj^* mice did not demonstrate the mineralization phenotype in their arterial blood vessels, and their enzymatic activity as well as plasma PP_i_ levels were between that of the wild-type and the *asj* mutant mouse.

**Implications and future directions**

This study shows that *Enpp1^asj^* mutant mice recapitulate the genetic, molecular and phenotypic features of humans with GACI, including autosomal recessive inheritance, inactivating mutations in the *Enpp1* gene, and profound mineralization of arterial blood vessels noted shortly after birth and resulting in early demise. The age of onset of the mineralization phenotype is notably earlier, and thereby closer to that in affected humans, than reported previously in mouse models of vascular mineralization. This is attributable, at least in part, to the administration of a special diet. Overall, the *asj* mouse serves as a model to study the pathomechanistic features of GACI, and provides a means to test pharmacologic approaches, such as bisphosphonates, towards treatment of this currently intractable disease.

### Evidence of aberrant mineralization

**Fig. 3.**
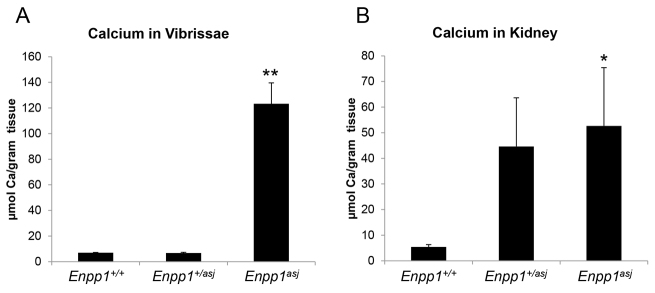
**Quantitation of mineralization by chemical assay of calcium in the dermal sheath of vibrissae and in the kidneys of mice in the range of 4–12 weeks of age kept on acceleration diet.** (A) Marked increase in mineralization is noted in the vibrissae of homozygous *asj* mice in comparison with wild-type or heterozygous animals (*n*=8). (B) Markedly increased mineralization in the kidneys of heterozygous and homozygous *asj* mice is noted (*n*=7–9). Statistical significance: *P<0.05 versus wild type; **P<0.001 versus wild type.

Histopathological examination of mice whose mothers were on the acceleration diet during pregnancy demonstrated extensive mineralization in the dermal sheath of vibrissae as well as in a number of internal organs when examined by hematoxylin and eosin (H&E) or Alizarin red stains (Fig. 1). Specifically, the dermal sheath of vibrissae was noted to be mineralized as early as at 4 weeks of age, and the mineralization progressed up to 12 weeks of age, the latest time point available for examination owing to early demise of the affected mice (Fig. 1B). The extent of mineralization was quantitated by chemical assay for calcium in the biopsies of muzzle skin containing the vibrissae in mice in the range of 4- to 12-weeks old, which showed a marked, up to 17.7-fold, increase in mineral content in *asj* mice compared with wild-type littermates (Fig. 3A). The heterozygotes were phenotypically and histopathologically normal, and the calcium content of the muzzle skin was low, the same as in normal wild-type mice (Fig. 3A).

**Fig. 4.**
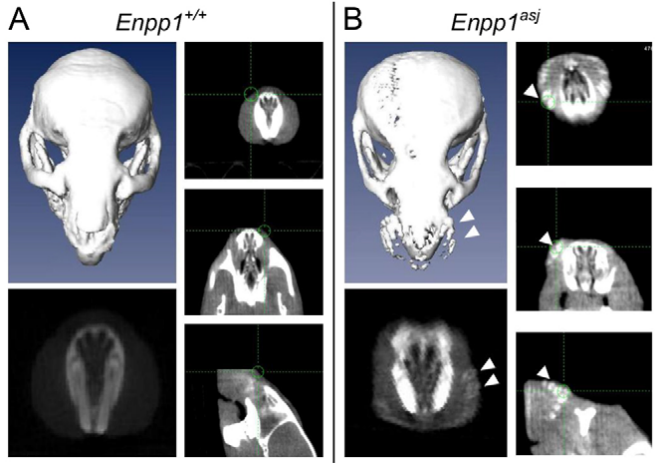
**Computed tomography demonstrating extensive mineralization of dermal sheath of vibrissae in a 7-week-old *asj* mouse in comparison with a wild-type littermate.** Single slices demonstrate evidence of mineralization (arrowheads in B), and computerized reconstruction reveals the mineral deposits in association with dermal sheath of vibrissae (upper left panel in B, arrowheads).

**Fig. 5.**
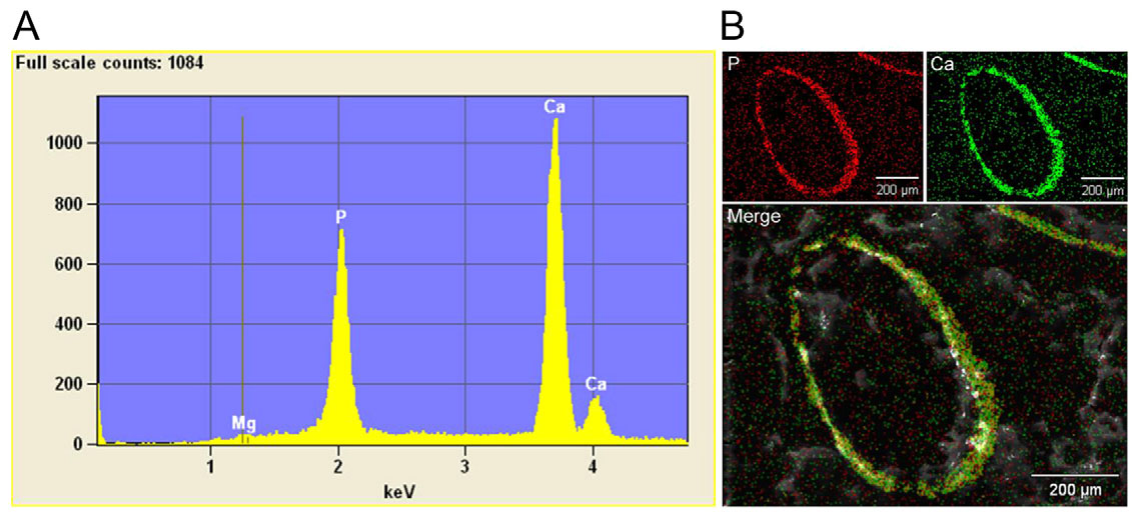
**Energy dispersive X-ray analysis of the mineral deposits in the dermal sheath of vibrissae in *asj* mice.** (A) Elemental composition analysis reveals the presence of calcium and phosphorus in ∼2:1 ratio. (B) X-ray topography of the distribution of phosphorus and calcium reveals colocalization, as demonstrated by merging the images.

The mineralization of the dermal sheath of vibrissae was also monitored noninvasively by computed tomography. At 7 weeks of age, the *asj* mice on the acceleration diet showed evidence of severe mineralization in the muzzle area, a finding that was not present in the wild-type mice (Fig. 4). The composition of mineral was further analyzed by energy dispersive X-ray (EDAX) of the deposits in the *asj* mouse vibrissae from histopathological sections (Kavukcuoglu et al., 2012). The analysis revealed calcium and phosphorus as the principal ions in ∼2:1 ratio, similar to that in endochondral bone (Fig. 5A). Topographic ‘radar’ mapping colocalized calcium and phosphorus, suggesting the presence of hydroxyapatite (Fig. 5B).

In addition to mineralization of the dermal sheath of vibrissae, extensive mineralization was noted in the aorta, as well as in the coronary and carotid arteries, and in the retina of the eye (Fig. 1B; Table 1). Also, blood vessels in the liver were mineralized, but no mineralization was noted in the liver parenchyma of the *asj* mice. No mineralization was noted in the dermal sheath of vibrissae, aorta or eyes in the heterozygote mice, but 2 of 13 heterozygotes showed mineralization in the heart. Only one wild-type mouse (1/13) showed evidence of mineralization in the eyes (Table 1). An interesting observation was that there was extensive mineralization in the kidneys of the *asj* mice kept on the acceleration diet (Fig. 1C). The mineralization affected primarily the medullary tubules as well as arcuate and renal arteries. Similar mineralization was noted in *Enpp1^+/asj^* heterozygous mice, as visualized by histopathology (Fig. 1C) and quantitated by direct chemical assay of calcium (Fig. 3B). Evidence of mineralization of the kidney of wild-type mice was also noted when kept on an acceleration diet, but to a much lower extent than in homozygous and heterozygous *asj* mice (Fig. 1C; Fig. 3B).

**Table 1.**
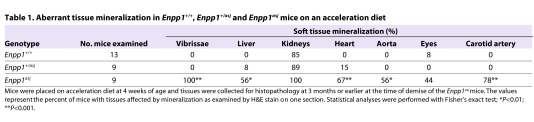
Aberrant tissue mineralization in *Enpp1^+/+^*, *Enpp1^+/asj^* and *Enpp1^asj^* mice on an acceleration diet

The *asj* mice on normal laboratory diet had a normal lifespan, and these *asj* mice had much less mineralization in the dermal sheath of vibrissae, as determined by histopathology, as compared with mice on the acceleration diet. In addition, the vascular mineralization phenotype was delayed until ∼5 months of age, as compared with early onset at 4 weeks when the mice were kept on the acceleration diet. Thus, the *asj* mice manifest with extensive mineralization of a number of connective tissues, and the extent of mineralization is clearly modulated by the diet.

### Genetic and molecular characterization

**Fig. 6.**
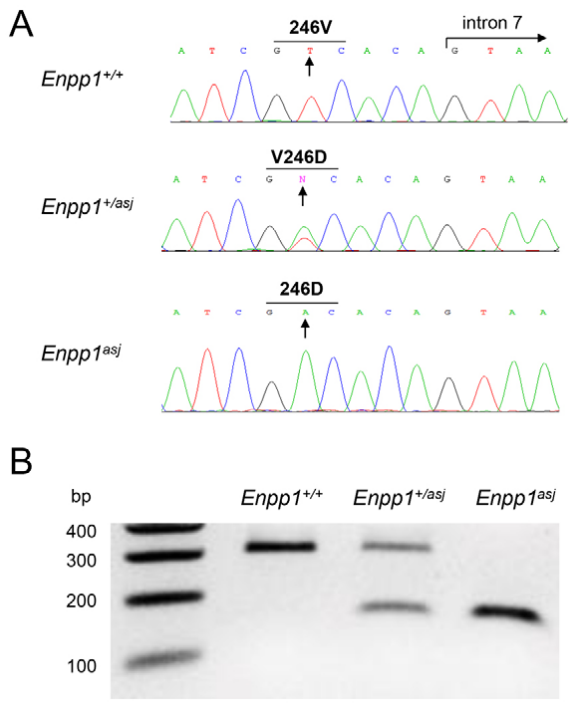
**Genotyping and mutation analysis of *asj* mice.** (A) Sequence analysis reveals a homozygous T-to-A nucleotide substitution (arrows) in *asj* mice, which results in substitution of valine 246 for an aspartic acid (p.V246D). (B) Taq^a^I restriction enzyme digestion of PCR products corresponding to exon 7 of the *Enpp1* gene reveals a 300 bp fragment representing the wild-type allele and a 150 bp fragment corresponding to the mutant allele.

Sequencing of the *Enpp1^asj^* mice confirmed that they were homozygous for a p.V246D substitution as a result of a T-to-A transversion mutation in position 771 within exon 7 of the *Enpp1* gene (Fig. 6A). This nucleotide substitution allows distinction of the corresponding wild-type and mutant *asj* alleles by restriction enzyme digestion with Taq^α^I, forming the basis of genotyping of these mice (Fig. 6B).

**Fig. 7.**
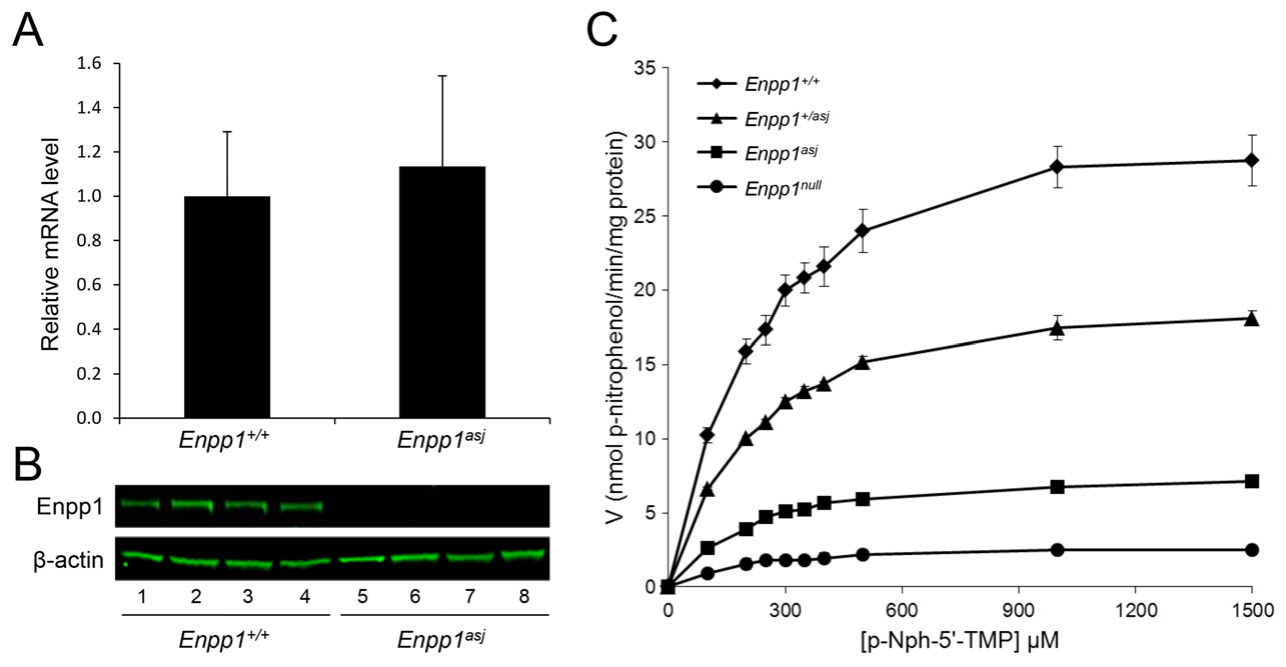
**Analysis of *Enpp1* mRNA, protein and enzymatic activity in *asj* mice.** (A) qPCR reveals similar relative mRNA levels in wild-type (*Enpp1^+/+^*) and mutant (*Enpp1^asj^*) mice (mean ± s.e.m.; *n*=7). (B) Western analysis of the liver protein with an anti-ENPP1-specific antibody demonstrates the presence of a band of 110 kDa in wild-type mice (lanes 1–4), whereas the protein is below the level of detection in *asj* mice (lanes 5–8). (C) Assay of ENPP1 enzymatic activity in the liver of wild-type, heterozygous mutants and homozygous *asj* mice (*n*=4), as well as of an *Enpp1^−/−^* knockout mouse (*n*=3). Note the markedly reduced activity in *asj* mice and intermediate activity in the heterozygotes in comparison with the wild-type mice.

To examine the consequences of the missense mutation in *Enpp1* at the mRNA and protein levels, quantitative PCR (qPCR) and western analyses were performed on four *asj* and four wild-type mice sacrificed at 4–12 weeks of age. The mRNA levels were not different in the liver of *asj* and wild-type mice by qPCR (Fig. 7A). However, isolation of the protein from liver with subsequent western analysis with an anti-ENPP1 specific antibody clearly revealed the presence of a band of the appropriate size, 110 kDa, in wild-type mice, but the level of protein was below the detection limit of the western analysis in *asj* mice (Fig. 7B). It should be noted that, although the antibody used is clearly specific for wild-type ENPP1, its precise epitope is not known and, consequently, our results do not exclude the possibility that it does not recognize the mutant protein. This possibility, however, is unlikely because the antibody used is polyclonal. The enzyme kinetics revealed that the ENPP1 isolated from the liver of *asj* mice was markedly reduced in activity (Fig. 7C). The Michaelis constant (*K*_m_) for the wild-type *Enpp1* mouse, as determined by a Hanes-Woolf plot, was 213.6±14.0 μM (mean ± s.e.m.), as compared with a *K*_m_ of 192.0±6.7 μM for *asj* mice (Fig. 7C) (*P*>0.05). However, the maximum rate of reaction (*V*_max_) for the wild-type enzyme was 33.4±2.1 nmol p-nitrophenol released/minute/mg protein versus 8.1±0.3 nmol in *asj* mice (*P*<0.01).

Because the *asj* mice demonstrated residual enzyme activity, yet western analysis showed the presence of little, if any, ENPP1 protein, two critical experiments were performed. First, the ENPP1 enzymatic activity was measured in heterozygous *asj* mice in comparison with wild-type and homozygous mutant mice. The results showed that the *K*_m_ in heterozygous mice was 203.8±13.1 μM. The *V*_max_ was 20.8±0.8 nmol, a 38% reduction compared with wild-type enzyme measured at linear range of reaction (*P*<0.05). Second, liver from *Enpp1^tm1Gdg^* knockout mice, developed by targeted ablation of the gene, was used for the corresponding enzyme assay (Sali et al., 1999). These mice showed a very low *V*_max_, 2.9±0.4 nmol, even less than in *asj* mice (*P*<0.05), suggesting that the *asj* mice might have some residual activity, and not be completely null (Fig. 7C).

To examine the consequences of reduced ENPP1 activity in *asj* mice, the concentrations of PP_i_ in plasma of wild-type, heterozygous mutant and homozygous mutant mice were determined in a three-step enzymatic reaction. As expected, the *asj* mice showed markedly reduced PP_i_ levels, ∼20% from the wild-type controls, with concomitant reduction in the PP_i_:P_i_ ratio, and the heterozygous mice showed intermediate levels (Table 2). The serum calcium and phosphorus levels and the corresponding Ca:P ratio were not statistically different in these three groups of mice (Table 2).

**Table 2.**
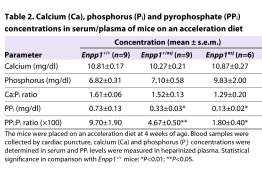
Calcium (Ca), phosphorus (P_i_) and pyrophosphate (PP_i_) concentrations in serum/plasma of mice on an acceleration diet

## DISCUSSION

In this study, we have extensively characterized a mutant *Enpp1^asj^* mouse as a model for GACI. This autosomal recessive disorder manifests with profound arterial mineralization; results of prenatal ultrasound can often suggest the possibility of this disease, which is then commonly diagnosed in the early postnatal period (Nitschke and Rutsch, 2012b; Rutsch et al., 2011). In most cases, the affected children die within the first 6 months of life as a consequence of vascular insufficiency causing end-organ damage. This disease in its classic form is caused by mutations in the *ENPP1* gene, which encodes ectonucleotide pyrophosphatase/phosphodiesterase 1 (ENPP1), also known as plasma cell membrane glycoprotein 1 (PC-1) (Ruf et al., 2005). This enzyme converts ATP to AMP and PP_i_, and this pathway is the main source of inorganic pyrophosphate. The extracellular PP_i_ plays a crucial role as an inhibitor of hydroxyapatite formation, whereas P_i_ promotes the formation of hydroxyapatite crystals. Thus, the mineralization is controlled by the PP_i_:P_i_ ratio as a result of activities of a number of enzymes, including ENPP1 and tissue nonspecific alkaline phosphatase (TNAP), as well as transporter proteins mediating the extracellular transport of P_i_ and PP_i_, including ankylosis protein (ANK) and type III sodium-dependent P_i_ co-transporter 1 (P_i_T1) (Mackenzie et al., 2012). Thus, a reduced PP_i_:P_i_ ratio in individuals with GACI as a result of reduced ENPP1 activity mechanistically leads to aberrant mineralization of extracellular connective tissues.

The *asj* mice examined in this study have several features that recapitulate GACI in humans. The hallmark of the disease, extensive arterial calcification, can be demonstrated in the *asj* mice as early as 4 weeks of age when the mice are on the acceleration diet, and the homozygous mice often die most likely as a consequence of aberrant mineralization of the aorta as well as of blood vessels in other tissues, including heart and carotid arteries. Notably, these mice also demonstrated mineral deposits in the retina of the eye as well as in the dermal sheath of vibrissae in the muzzle skin. Similar to individuals with GACI, the plasma concentration of PP_i_ was markedly reduced (<20% of the control mice) and the heterozygotes demonstrated intermediate levels. It should be noted that the heterozygous littermates did not demonstrate vascular mineralization, with the exception of the kidneys, and were indistinguishable from the wild-type controls, attesting to the autosomal recessive mode of the *asj* mutant allele. In this context, it should be noted that currently there is no evidence for modulation of the *Enpp1^asj^* mutation by other genes, such as *Abcc6* in PXE mice (Klement et al., 2005) or in some inbred strains of mice (KK/HlJ, DBA/2J and C3H/HeJ) (Berndt et al., 2013) associated with vascular mineralization.

The *asj* mouse was developed at The Jackson Laboratory as part of the ENU mutagenesis program (http://mousemutant.jax.org/articles/mmrmutantasj.html). The phenotypic features of these mice include stiffening of the joints [hence ‘ages with stiffened joints’ (*asj*)], and these mice were shown to harbor a missense mutation, p.V246D, in the *Enpp1* gene. Initial necropsies of these mice at 7 months of age revealed periarticular mineralization of the ligaments and mineralization of the dermal sheath of vibrissae. Previously, mutations affecting the *Enpp1* gene have been described in a mutant mouse, ‘tip toe walking’ (*ttw/ttw*), shown to harbor a stop codon mutation in the *Enpp1* coding sequence (Okawa et al., 1998). Similarly, *Enpp1^tm1Gdg^* knockout mice exhibit abnormalities similar to those in *ttw/ttw* mice (Sali et al., 1999). Furthermore, another *Enpp*1 mutant mouse, with a p.C397S missense mutation, has been characterized by low bone mineral density, crystal-related arthropathy and vascular calcification (Babij et al., 2009). Characterization of these mice has largely focused on alterations in bone mineralization in long bones, the calvariae and periarticular, as well as perispinal soft tissue mineralization. Although arterial calcification was documented in some of these previously described mice, the changes were frequently not noted to be present until 16–22 weeks of age (Babij et al., 2009; Mackenzie et al., 2012). In *asj* mice, the mineralization was noted to occur as early as 4 weeks of age, with an early demise at ∼6 weeks of birth when on the acceleration diet. Consequently, this mouse model accurately recapitulates features of GACI, a disease that is usually lethal within the first 6 months of life. This difference in the age of onset of mineralization can be attributable, at least in part, to the special diet that was used in our study to accelerate the mineralization process. This ‘acceleration diet’ consists of increased phosphate and reduced magnesium in comparison with the standard mouse laboratory diet (Jiang and Uitto, 2012; Li and Uitto, 2010). Specifically, the phosphate concentration was increased twofold (8.5 mg/g of food), and the magnesium content was reduced to 20% of the control (0.4 mg/g of food). All the mice were placed on this experimental diet at weaning at 4 weeks of age but, in addition, the mothers during the pregnancy and lactation were on this special diet. We previously showed that this diet also accelerates the aberrant mineralization noted in *Abcc6^tm1Jfk^*-null mice, a model of another aberrant mineralization disorder, pseudoxanthoma elasticum (Jiang and Uitto, 2012). It should be noted, however, that this diet does not cause any mineralization in the arterial vessels, the eyes or in the vibrissae in wild-type control mice or in heterozygotes of mutations in either *Enpp1* or *Abcc6*.

An interesting observation was extensive mineralization in the kidneys of homozygous and heterozygous *asj* mice, and wild-type control mice also showed some evidence of aberrant mineralization when the mice were placed on the experimental acceleration diet. The observed mineralization of kidneys in the heterozygous mice differs from that in humans in that heterozygous carriers of *ENPP1* mutations do not show any evidence of mineralization. Similar findings of nephrocalcinosis have previously been noted in mice with a high phosphorus-containing diet, similar to individuals with hyperphosphatemia (Markowitz and Perazella, 2009). The increased mineralization in the kidney suggests that this might be a process determined by a more complex genetic background in which ENPP1 plays a role but is not the sole contributor.

In their classic forms, GACI and PXE are two clinically distinct conditions, GACI manifesting with extensive arterial calcification at birth leading to early demise of the affected individuals, whereas the clinical manifestations and tissue mineralization in PXE is of late onset and slowly progressive (Nitschke and Rutsch, 2012b; Uitto et al., 2010). However, recently, individuals with features of early GACI with development of PXE, with characteristic skin findings, have been noted (Le Boulanger et al., 2010; Li et al., 2012). In addition, a subset of individuals with GACI has been recently shown to harbor mutations in the *ABCC6* gene, instead of *ENPP1* (Nitschke and Rutsch, 2012b). These observations suggest the presence of common pathomechanistic pathways leading to aberrant tissue mineralization (Nitschke and Rutsch, 2012a). Although the pathomechanistic details of tissue mineralization particularly in the case of PXE are currently unknown, it should be noted that the PP_i_ levels in plasma of *Abcc6^−/−^* mice are not altered (Q.L. et al., unpublished). Finally, there are a number of additional heritable aberrant mineralization disorders resulting in calcium deposits in the skin, including normophosphatemic and hyperphosphatemic familial tumoral calcinosis, and arterial calcification with CD73 deficiency, each due to mutations in different genes (Li and Uitto, 2013). The presence of hydroxyapatite crystal deposition in these phenotypically diverse conditions attest to the complex mineralization/anti-mineralization network required for normal homeostasis. Dissection of the pathomechanistic details leading to aberrant mineral deposition in these single-gene disorders will assist us in the development and testing of efficient treatment modalities. In this context, the *asj* mice could well serve as a model system to study potential treatment modalities for GACI under genetically and environmentally controlled conditions. In this regard, a few studies have reported improvement in some patients with GACI with treatment with bisphosphonates, but controlled studies attesting to the efficacy of this treatment strategy are lacking (Edouard et al., 2011; Ramjan et al., 2009). Furthermore, this mouse model could be used to test other compounds, such as pyrophosphate and sodium thiosulfate, which have been suggested to counteract vascular mineralization (Hayden and Goldsmith, 2010; Ning et al., 2013; O’Neill et al., 2011). Collectively, the ability to carefully dissect and analyze *asj* mice could provide an enhanced clinical understanding of GACI and contribute towards improved treatment.

## MATERIALS AND METHODS

### Animals and diet

*Enpp1^asj^* mice on a C57BL/6J background were obtained from The Jackson Laboratory (Bar Harbor, ME) (http://mousemutant.jax.org/articles/mmrmutantasj.html). *Enpp1^+/+^* and *Enpp1^asj^* mice were generated from heterozygous matings. Mice were maintained either on standard laboratory diet (Laboratory Autoclavable Rodent Diet 5010; PMI Nutritional International, Brentwood, MO) or fed an ‘acceleration diet’ (Harlan Teklad, Rodent diet TD.00442, Madison, WI), which we have previously shown to accelerate the ectopic mineralization in *Abcc6^−/−^* mice; this diet is enriched in phosphorus and has reduced magnesium content (Jiang and Uitto, 2012). The mice were maintained under standard conditions at the Animal Facility of Thomas Jefferson University, and all protocols were approved by the Institutional Animal Care and Use Committee of Thomas Jefferson University. Proper handling and care were followed according to the Animal Welfare Policies of the Public Health Service.

### Genotyping and gene sequencing

A primer pair with sequences 5′-TGATCTGCATCCTGGGATAA-3′ and 5′-TAAGGAAAGACCAATTGCAGA-3′ was used to amplify exon 7 of the *Enpp1* gene. To identify the wild-type and *Enpp1^asj^* mutant alleles, PCR products were digested with Taq^α^I (New England BioLabs, Ipswich, MA), producing a band of either 300 bp or 150 bp, respectively.

Gene sequencing was performed at the Kimmel Cancer Center Nucleic Acid Facility at Thomas Jefferson University using the BigDye Terminator v.3.1 Cycle Sequencing Kit (Applied Biosystems, Foster City, CA). Products were analyzed on the 3730 DNA Analyzer (Applied Biosystems) and the results were visualized with Chromas software (Technelysium, South Brisbane, Australia).

### Quantitative PCR

qPCR was performed using an ABI Prism 7000 sequence detection system (Applied Biosystems) with Power SYBR Green PCR Master Mix, as described previously (Li et al., 2007). *Enpp1* primers had sequences 5′-GCCAAAGACCCCAACACCTACAAA-3′ and 5′-ACAGGTCTCCTGGAAATCCAGACA-3′. The amount of *Enpp1* mRNA per sample was quantified and normalized to *Gapdh* mRNA, and relative expression levels were calculated by the ΔΔCt method. The dissociation curve was generated with Dissociation Curve software, version 1.0 (Applied Biosystems), to determine reaction specificity.

### EDAX analysis and topographic mapping

For energy dispersive X-ray analysis (EDAX), paraffin sections of muzzle skin containing mineral deposits were mounted onto carbon carriers and analyzed for elemental composition with a JEOL-T330A scanning electron microscope (JEOL Ltd, Tokyo, Japan) fitted with an EDAX microanalysis analyzer. X-ray topographic maps of calcium and phosphorus were collected with Thermo Scientific NSS software, version 2.3 (Swedesboro, NJ).

### Western blot

Liver lysates were prepared by homogenizing tissues in lysis buffer containing PMSF (Sigma, St Louis, MO), phosphatase inhibitor cocktail (Sigma), protease inhibitor cocktail (Thermo Scientific, Rockford, IL) and 8M urea (Fisher, Pittsburgh, PA) in RIPA buffer (Sigma). A BCA kit (Thermo Scientific) was used to determine the protein concentration of all lysates.

Protein, ∼90 μg per lane, was loaded in an 8% gel (Thermo Scientific) for SDS-PAGE, and the proteins were then transferred to a PVDF membrane. The membrane was blocked in 5% milk/TBS + 0.1% Tween-20 at room temperature for 1 hour and then incubated with anti-ENPP1 primary antibody (Cell Signaling, Danvers, MA), 1:500 in dilution buffer (2% milk/TBS + 0.1% Tween-20) at 4°C overnight. To visualize the signal, the membrane was incubated in anti-rabbit secondary antibody (LI-COR, Lincoln, NE) in dilution buffer for 1 hour at room temperature and then scanned with an Odyssey Infrared Imager (LI-COR). The membrane was then stripped and reprobed with anti-β-actin antibody (Bioorbyt, San Francisco, CA) 1:750 in dilution buffer.

### Enzyme activity assay

ENPP1 activity in wild-type, heterozygous and *asj* mouse liver, as well as in *Enpp1^tm1Gdg^* knockout mice (kindly provided by Dr Robert Terkeltaub, University of California, San Diego, CA), was determined using the substrate thymidine 5′-monophosphate p-nitrophenyl ester (p-Nph-5′-TMP, Sigma). Total proteins were extracted from whole liver with lysis buffer containing 50 mM HEPES, 0.1 mM EGTA, 0.1 mM EDTA, 120 mM NaCl, 0.5% NP-40, pH 7.5, PMSF, and complete protease inhibitor (Roche) (Babij et al., 2009). A BCA kit (Pierce) was used to determine the protein concentration of lysates. Protein lysates were diluted with assay buffer (50 mM Tris-HCl, pH 9.5, and 250 mM NaCl in water) to 100 ng/μl. In 96-well plates, 50 μl of p-Nph-5′-TMP (diluted with assay buffer to nine different concentrations) was added to 50 μl of protein lysate. All samples were tested in duplicate. The samples were then incubated at 37°C, and absorbance (400 nm) was measured with a microplate reader (Bio-Rad 800) every 5 minutes for up to 30 minutes to ensure linearity of the reaction. A molar extinction coefficient of the reaction product, p-nitrophenol, of 18.4×10^3^ M^−1^ cm^−1^ was used in determination of enzyme kinetics. Enzyme activity was expressed as nmol p-nitrophenol released per minute per mg of protein.

### Quantification of calcium and phosphate

To quantify the calcium deposition in the dermal sheath of mouse vibrissae, and the kidneys, muzzle skin and kidney were harvested and decalcified with 0.15 N HCl for 48 hours (skin) or with 0.6 N HCl for 1 week (kidney) at room temperature. The calcium content in these samples as well as in serum was determined colorimetrically by the *o*-cresolphthalein complexone method [Calcium (CPC) Liquicolor; Stanbio Laboratory, Boerne, TX]. The phosphate content of serum was determined with Malachite Green Phosphate Assay kit (BioAssay Systems, Hayward, CA). The values for calcium and phosphate were normalized to tissue weight.

### Inorganic pyrophosphate assay

PP_i_ was measured by an enzymatic assay using uridine-diphosphoglucose (UDPG) pyrophosphorylase as previously described (Lomashvili et al., 2005; O’Neill et al., 2010), with modifications. Heparinized plasma samples (20 μl; 1:4 dilution) were heated at 65°C for 10 minutes, followed by three different assays performed on each sample: (1) no addition of PP_i_ standard, (2) pre-incubation with 0.35 U pyrophosphatase at 37°C for 1 hour, and (3) addition of 3 μM PP_i_. Samples were then added to 100 μl of reaction buffer that contained 5.2 mM Mg Acetate, 57 mM Tris Acetate (pH 7.8), 4 μM NADP, 7.5 μM UDPG, 18.6 μM glucose-1, 6-diphosphate, 0.14 U UDPG pyrophosphorylase, 2.5 U phosphoglucomutase, 0.4 U glucose-6-phosphate dehydrogenase, and 0.02 μCi [^3^H]UDPG. After a 30-minute incubation at 37°C, 200 μl of 4% activated charcoal was added to each sample with occasional stirring to bind residual UDPG. After centrifugation, the radioactivity in 100 μl of supernatant was counted. The plasma [PP_i_] was determined as (CPM1-CPM2)/(CPM3-CPM1)×3 μM.

### Histopathological analysis

Muzzle skin and internal organs from euthanized mice were fixed in 10% phosphate-buffered formalin, routinely processed, and embedded in paraffin. Tissues were sectioned (6 μm) and stained with H&E and Alizarin red using standard procedures. Slides were examined under light microscopy for mineralization and other lesions by an experienced veterinary pathologist (J.P.S.).

### Small-animal computed tomography (CT scan)

*Enpp1* wild-type and *asj* mice were examined for mineralization at 7 weeks of age by CT scan, as described (Le Corre et al., 2012). Briefly, mice were anesthetized with a xylazine-ketamine-acetopromazine cocktail (160 μl per 25 g body weight of 10 mg/kg xylazine, 200 mg/kg ketamine, 2 mg/kg acetopromazine) and then scanned with a MicroCAT II (ImTek Inc., Oak Ridge, TN). A 3-dimensional facial rendering was created for each mouse using Amira software, version 3.1 (Visualization Sciences Group, Burlington, MA).

### Statistical analysis

The comparisons in different groups of mice were completed using two-sided Kruskal-Wallis nonparametric tests. The Kruskal-Wallis test is comparable to one-way analysis of variance, but without the parametric assumptions. Fisher’s exact test was used to determine the difference between proportions in mineralization phenotypes in mice. All statistical computations were completed using SPSS version 15.0 software (SPSS Inc., Chicago, IL).
